# Molecular Dynamics Analysis of Apolipoprotein-D - Lipid Hydroperoxide Interactions: Mechanism for Selective Oxidation of Met-93

**DOI:** 10.1371/journal.pone.0034057

**Published:** 2012-03-30

**Authors:** Aaron J. Oakley, Surabhi Bhatia, Heath Ecroyd, Brett Garner

**Affiliations:** 1 Illawarra Health and Medical Research Institute, University of Wollongong, New South Wales, Australia; 2 School of Chemistry, University of Wollongong, New South Wales, Australia; 3 Neuroscience Research Australia, Sydney, New South Wales, Australia; 4 School of Biological Sciences, University of Wollongong, New South Wales, Australia; Consejo Superior de Investigaciones Cientificas, Spain

## Abstract

**Background:**

Recent studies suggest reduction of radical-propagating fatty acid hydroperoxides to inert hydroxides by interaction with apolipoprotein-D (apoD) Met93 may represent an antioxidant function for apoD. The nature and structural consequences of this selective interaction are unknown.

**Methodology/Principal Findings:**

Herein we used molecular dynamics (MD) analysis to address these issues. Long-timescale simulations of apoD suggest lipid molecules are bound flexibly, with the molecules free to explore multiple conformations in a binding site at the entrance to the classical lipocalin ligand-binding pocket. Models of 5s- 12s- and 15s-hydroperoxyeicosatetraenoic acids were created and the lipids found to wrap around Met93 thus providing a plausible mechanism by which eicosatetraenoic acids bearing hydroperoxides on different carbon atoms can interact with Met93 to yield Met93 sulfoxide (Met93SO). Simulations of glycosylated apoD indicated that a second solvent exposed Met at position 49 was shielded by a triantennerary N-glycan attached to Asn45 thereby precluding lipid interactions. MD simulations of apoD showed B-factors of the loop containing Met93SO were higher in the oxidized protein, indicating increased flexibility that is predicted to destabilize the protein and promote self-association.

**Conclusions/Significance:**

These studies provide novel insights into the mechanisms that may contribute to the antioxidant function of apoD and the structural consequences that result if Met93SO is not redox-cycled back to its native state.

## Introduction

Apolipoprotein-D (apoD) is a 29 kDa glycoprotein member of the lipocalin family that comprises an eight-stranded antiparallel β-barrel flanked by an α-helix [1]. The β-barrel encloses a hydrophobic ligand pocket that binds arachidonic acid and progesterone with high affinity [1], [2]. The eicosanoids 12-hydroxyeicosatetraenoic acid (12-HETE) and 5,15- dihydroxyeicosatetraenoic acid have also been shown to bind to apoD albeit with lower affinity [3]–[5]. In addition to the classical lipocalin pocket [6], apoD may also interact with lipids via a cluster of exposed hydrophobic residues residing in three of its extended loops [1]. These exposed residues generate a hydrophobic surface region close to the open end of the binding pocket that facilitates apoD association with high-density lipoprotein (HDL) particles and is also thought to permit insertion of apoD into cellular lipid membranes [1].

Although apoD is well known to bind lipids and thereby thought to play a role in lipid transport in the brain and in plasma [7], it has also more recently been associated with lipid antioxidant protection. This has been clearly demonstrated using apoD null and apoD over-expressing mice [8] and in an ageing *Drosophila* model [9]. In these studies apoD overexpression protected against insults that promote cerebral lipid peroxidation whereas deletion of the APOD gene increased susceptibility to oxidative stress. This antioxidant function might also explain why apoD levels are increased in the human brain in association with ageing and Alzheimer's disease [10]–[12] as it could serve as a protective response to combat the increased levels of lipid peroxidation that are know to occur under these conditions [13], [14].

We have recently shown that apoD catalyzes the reduction of potential free-radical generating lipid hydroperoxides (L-OOHs) to relatively inert lipid hydroxides (L-OHs) via a selective interaction with apoD Met_93_ [15]. As a consequence of this reaction Met_93_ is selectively converted to MetSO [15]. We have proposed that this reduction of L-OOH to L-OH competes with transition metal-catalyzed Fenton-type reactions that could otherwise generate chain-propagating radicals from L-OOHs [15]. Although this pathway may contribute to the apoD antioxidant mechanism, it is presently unclear how different lipid hydroperoxides may interact selectively with apoD Met_93_ when high affinity lipid binding within the apoD ligand pocket is known to be selective [2]. In addition, details of the structural changes that may occur in apoD as a result of Met_93_SO generation remain to be defined.

The aim of the present study was therefore to use MD simulations and molecular modeling approaches to investigate the interactions of arachidonic acid and its lipoxygenase-derived HpETE products with apoD and to assess the potential structural consequences of apoD Met_93_ oxidation.

## Materials and Methods

### ApoD molecular dynamics simulations and modelling

In order to understand the structural basis for the behavior of apoD, modeling and MD simulations were performed. All MD trajectories were calculated using NAMD 2.8 [16]. For all simulations of apoD, models were embedded in a water box with overlapping water molecules removed and sodium ions added to ensure that the systems had no net charge. Temperature control at 310 K was maintained with Langevin dynamics (damping constant: 5 ps^−1^) applied to non-hydrogen atoms. Periodic boundary conditions were used with the Nosé-Hoover Langevin piston method (piston period 100 fs, decay rate 50 fs) to maintain a constant pressure of 1.013 Bar. The Particle-mesh Ewald algorithm was used to account for long-range electrostatic effects (grid resolution<1 Å). All other non-bonded interactions were calculated using a switching function to smooth interactions to zero between 10 and 12 Å. The integration timesteps were 1, 2, and 4 fs for bonded, nonbonded, and long-range electrostatic interactions respectively. Every system was initially equilibrated for 100 ps, after which the MD run was extended as described below. Coordinates were saved every 1 ps for analysis.

To determine the effects of Met_93_ oxidation on stability of apoD, 50 ns simulations of apoD and ApoD-Met_93_SO without bound ligands were calculated. The starting configuration for these calculations was the published structure of ligand-free apoD (PDB: 2HZR), with missing residues modeled, selenoMet residues changed to Met and any mutated residues reverted to wild-type. To assess the ability of the simulations to reproduce native protein-ligand conformations, a 100 ns simulation of the complex of apoD with progesterone was performed using the crystal structure of this complex (PDB: 2HZQ) for the starting configuration. To understand the behavior of lipid molecules bound to apoD, four 200 ns runs were produced with arachidonic acid modeled in the binding site in different extended conformations. Low energy conformations were used to model arachidonate metabolites 5s-, 12s- and 15s-HpETE bound to apoD. Finally, a 50 ns simulation of the apoD-progesterone complex with experimentally determined glycosylation was performed. In all cases the CHARMM protein force field [17] with backbone 2D dihedral energy correction (CMAP) [18] was used. Parameters for MetSO were adapted from parameters for DMSO [19]. The CHARMM general force-field [20] and carbohydrate derivative forcefield [21] was used for progesterone and glycosylations respectively. Trajectory data were analyzed using VMD [22]. Protein-ligand interaction energies were estimated using the generalized Born implicit solvent model [23]. Energies of isolated protein and ligand entities were calculated and subtracted from the energy of the combined system. For modeling HpETE compounds, parameters were taken from the CHARMM General Force Field (CGenFF) [20] or were derived from *ab inito* calculations on the model compound 2-hydroperoxypropane using Gaussian09 [24], performed at the MP2/6-31G* level of theory.

### ApoD analysis by circular dichroism (CD)

Recombinant forms of human apoD (NM_001647.3) containing a C-terminal linker, FLAG-tag and poly His tail (SGGGGSDYKDDDDKHHHHHH) were synthesized in HEK293 cells (American Type Culture Collection (ATCC), Catalogue No. CRL-1573, Manassas, VA, USA) and purified using a Ni-HiTrap column as described previously [15]. To generate apoD containing Met_93_SO, wild type apoD (0.5 mg/ml) was incubated in PBS containing 15s-HpETE (0.05 mg/ml) for 4 h. The protein fraction was precipitated with 9 volumes of ice-cold ethanol for 1 h at −20° and the pellet re-suspended in 20 µl of PBS in preparation for CD analysis. Far UV CD spectra for apoD and its mutants (apoD, M_49_-A, M_93_-A, M_157_-A) were acquired using Jasco J-810 CD spectropolarimeter at 22°C. Protein samples were prepared to final concentration of 0.1 mg/ml in PBS and spectra recorded using 0.1 cm quartz cuvette. Measurements were taken from 195–260 nm using a scan speed of 100 nm/min, 1 nm bandwidth, 1 s response time and data pitch of 0.1 nm. Each spectrum is an average of 6 scans and corrected by subtraction of the PBS spectrum acquired under similar conditions.

## Results

### ApoD structure and position of Met residues

**Figure 1:**
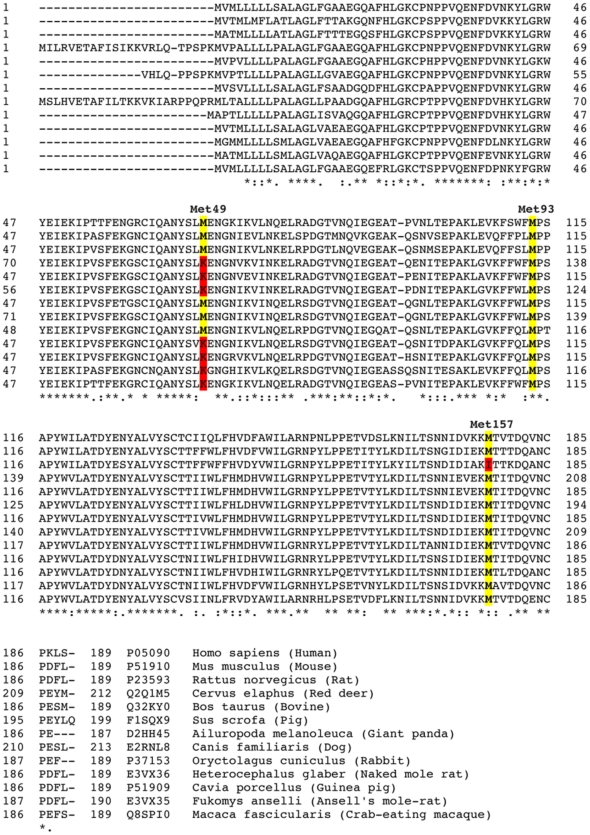
ApoD Clustal alignment. ApoD sequences were aligned using the UniProt Consortium Clustal alignment tool for the listed mammalian species. The positions of the three human Met residues are annotated (using the human residue numbering without the 20 amino acid signal peptide). Conserved Met residues are highlighted in yellow with substituted amino acids highlighted in red.

**Figure 2:**
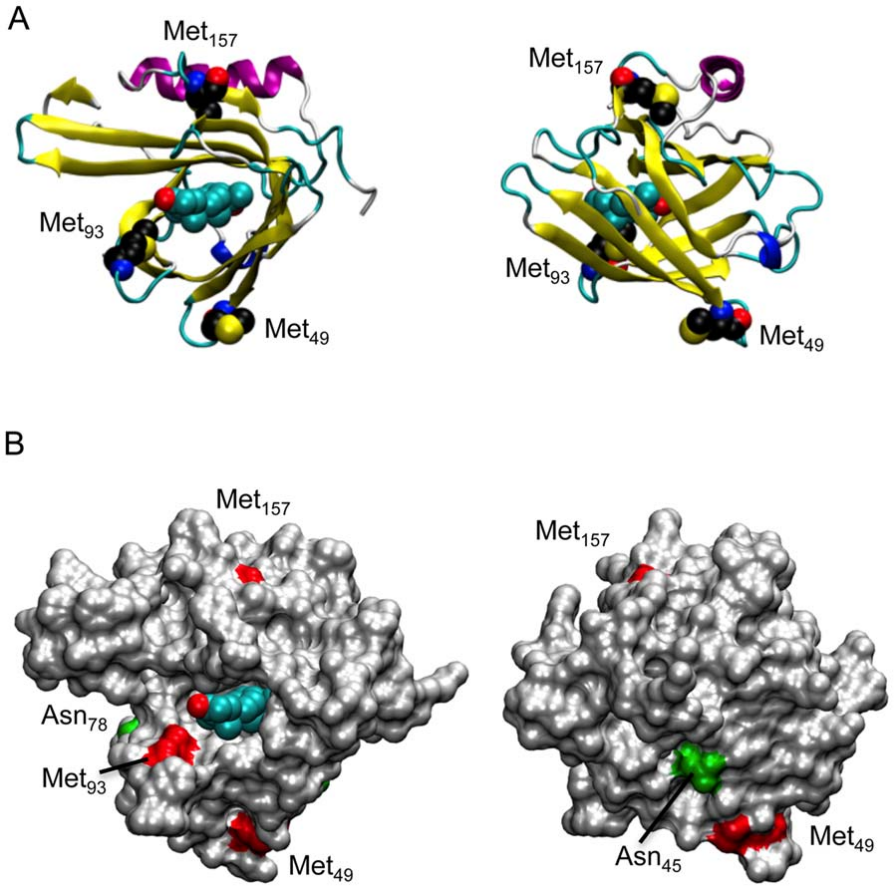
ApoD structure. (*A*) Orthogonal views of apoD in cartoon form with progesterone bound. The ligand and methionine residues are represented as van der Waals spheres. (*B*) Same as (*A*) but with protein represented as a solvent-accessible surface. Methionine residues (red patches) and glycosylated asparagine residues (green patches) are indicated.

We first examined apoD amino acid sequence across a variety of mammalian species. ApoD contains three Met residues that are highly conserved (Figure 1). In humans, apoD Met residues are Met_49_, Met_93_ and Met_157_. Met_93_ is located in a hydrophobic region at the opening of the ligand-binding pocket and is clearly exposed to the surface (Figure 2). Met_49_ is also exposed to the surface, residing within a β-strand stretch four amino acids C-terminal to one of the two N-linked glycosylation sites (Asn_45_). Met_157_ appears to be largely buried beneath a surface-accessible α-helix (Figure 2).

### MD simulation of apoD interactions with progesterone and arachidonic acid

In a recent study we reported that apoD exhibits a lipid antioxidant activity that we propose is due to the direct interaction of the apoD Met_93_ side chain with lipid hydroperoxides such as 5s-, 12s- and 15s-HpETEs [15]. Previous work indicated the high affinity binding of lipids within the apoD ligand binding pocket is selective and it was therefore unclear how different lipid hydroperoxides may interact with Met_93_.

**Figure 3:**
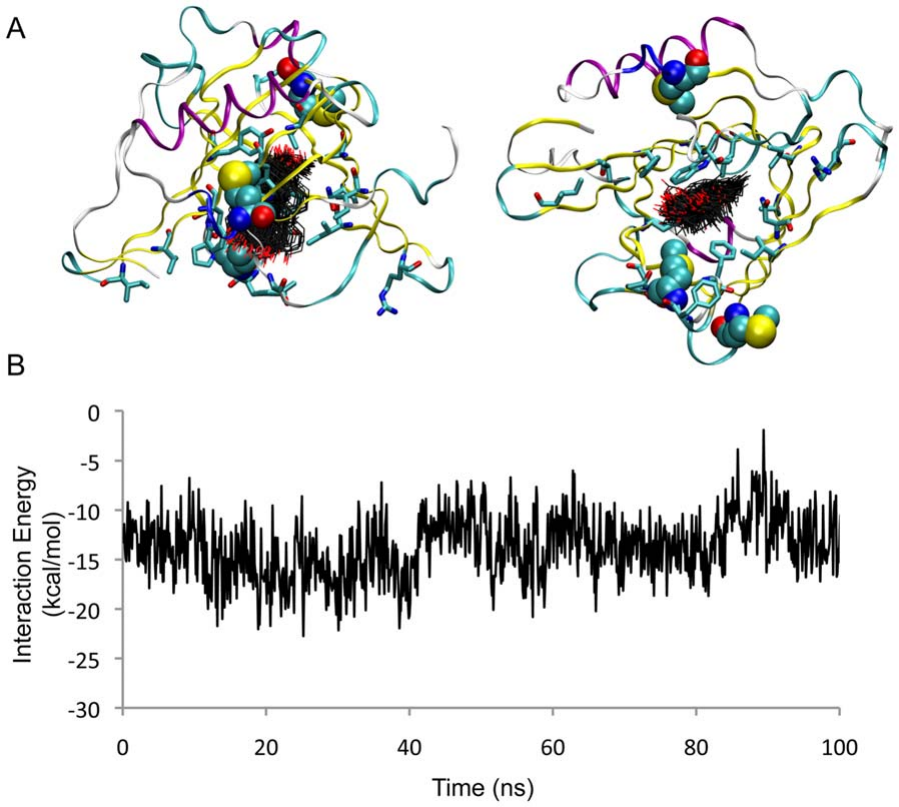
MD simulation of apoD with progesterone. (*A*) Orthogonal views of apoD in ribbon form with ligand-binding residues shown (cyan carbon atoms). The conformation of progesterone, sampled at 1 ns intervals, is shown (black carbon atoms). Methionine residues are represented as van der Waals spheres. (*B*) Interaction energy is plotted as a function of time.

We first assessed the interaction of progesterone with apoD as crystallography studies indicate this lipid forms a stable complex within the ligand-binding pocket [1]. In a 100 ns simulation of progesterone bound to apoD, the ligand remained close to the crystallographic conformation, with binding energies fluctuating between −1 and −23 kcal/mol (Figure 3). The RMSD of ligand atoms with respect to the crystallographically observed conformation was between 2 and 3.5 Å. A small degree of lateral sliding of the ligand in the pocket was observed in our MD simulation. All crystallographically observed contacts between progesterone and apoD were maintained over 100 ns.

**Figure 4:**
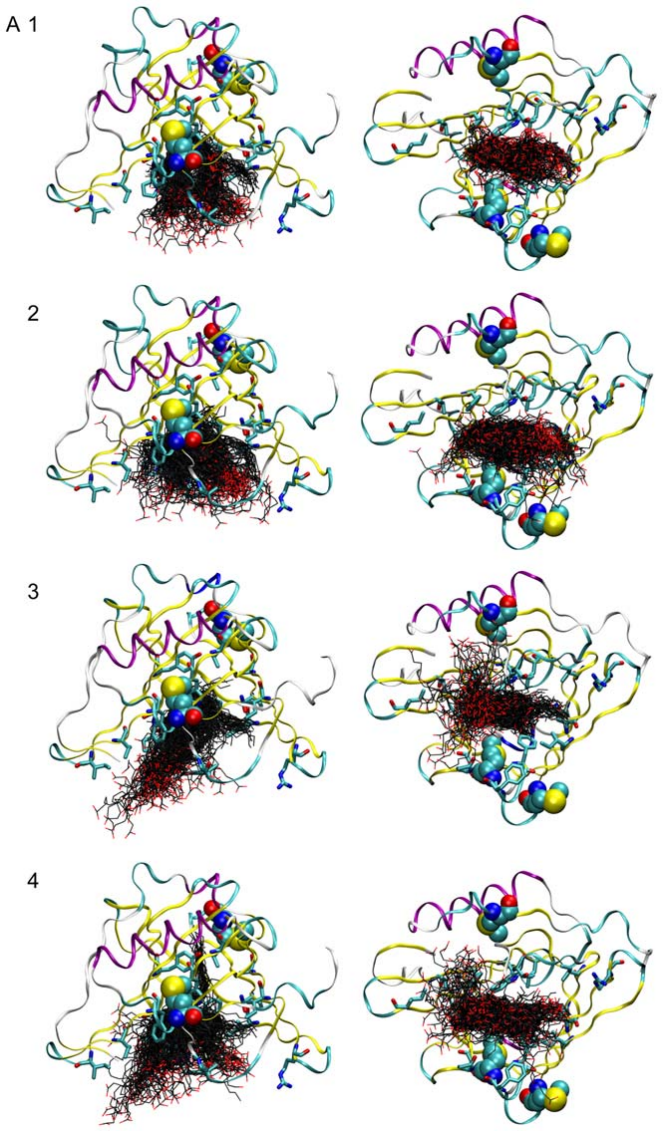
MD simulation of apoD with arachidonate. Orthogonal views of apoD in ribbon form with ligand-binding residues shown (cyan carbon atoms). The conformation of arachidonate, sampled at 1 ns intervals, is shown for simulations 1 to 4 (black carbon atoms). Methionine residues are represented as van der Waals spheres.

**Figure 5:**
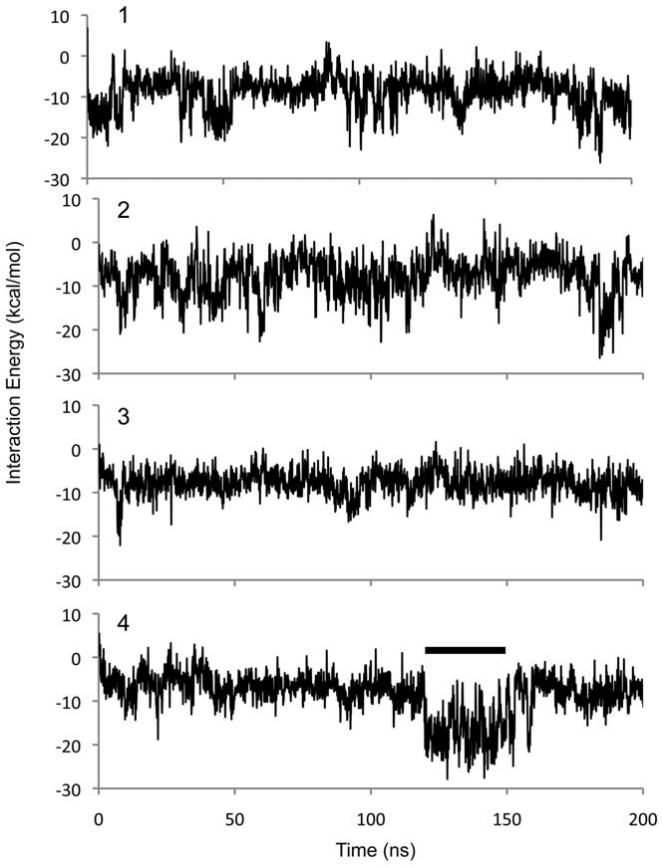
Interaction energy for MD simulation of apoD with arachidonate. Interaction energy of the four runs shown in Figure 4 are plotted as a function of time. The conformations in Figure 4 simulation 4 used for modeling of HpETE are indicated by a bar.

We next examined the interaction of arachidonic acid with apoD. Four simulations of apoD with bound arachidonic acid, totaling 800 ns, indicated that the principal binding site is at the opening of the ligand binding pocket and that this hydrophobic surface appears to behave as a “greasy slide”. In none of the four simulations, did arachidonate converge on a particular bound conformation (Figure 4). Instead, arachidonate continuously explores conformational space. In the majority of conformations sampled, arachidonate is extended length-ways across the binding site or is partly curled up in the binding site. In simulation 3, arachidonate became partly unbound but re-entered the binding site. In simulation 4, the tail of arachidonic acid became transiently buried in the binding site in a manner reminiscent of progesterone. Also in simulation 4, arachidonate adopted a conformation with low energy of interaction lasting approximately 30 ns (Figure 5), in which the carboxylic acid group of the fatty acid engaged in a salt bridge interaction with Arg_62_, with the remainder of the molecule lying in the binding site in a crescent-shaped conformation wrapped around Met_93_. This population of low-energy arachidonate conformations was used to model binding of 5s-, 12s- and 15s-HpETE to apoD.

### Modeling of HpETE interaction with apoD

**Figure 6:**
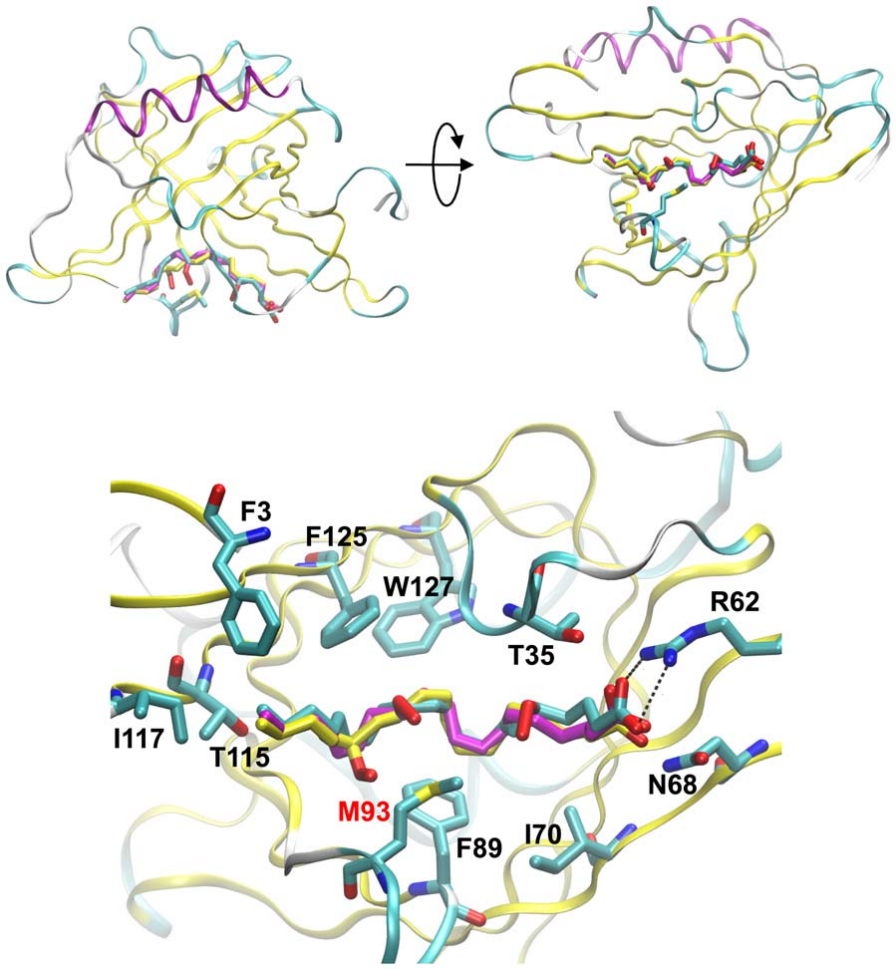
Models of apoD interaction with 5-, 12- and 15-HpETE. ApoD with 5- (cyan carbon atoms) 12- (magenta carbon atoms) and 15-HpETE (yellow carbon atoms) modeled in the binding site. Met_93_ is shown. The expanded view illustrates the close proximity of the Met_93_ side chain to the HpETE peroxide moiety. The salt bridges between Arg_62_ and the fatty acid are also indicted in the expanded view as dashed lines.

The absence of appropriate parameters for lipid hydroperoxides in the CHARMM force-field currently precludes simulation of this class of molecule with apoD. Nevertheless, in order to understand how L-OOHs might bind to apoD, the peroxidized arachidonate products 5s-, 12s-, and 15s-HpETE were modeled based on the most stable low-energy conformation observed for arachidonate. The models are shown overlaid in Figure 6. These models suggest that the L-OOH molecules wrap around Met_93_ thus providing a plausible mechanism by which eicosatetraenoic acids bearing peroxides on different carbon atoms can interact with Met_93_ and give rise to Met_93_SO.

### MD simulations of glycosylated apoD – Met_49_ is shielded

**Figure 7:**
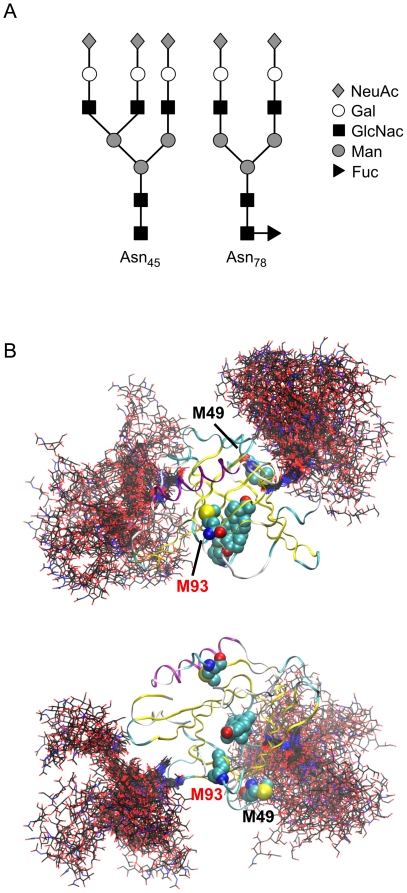
MD simulation of glycosylated apoD. (*A*) Schematic representation of glycosylation patten found at Asn_45_ and Asn_78_. Symbols are: sialic acid (diamonds), mannose (filled circles), galactose (open circles), N-acetylglucosamine (squares) and fucose (triangle). (*B*) Orthogonal views of apoD in ribbon form with progesterone and methionine residues are represented as van der Waals spheres. The conformation of the glycosylations, sampled at 1 ns intervals, are shown (black carbon atoms).

Data derived from our previous studies [15] and the simulations above clearly indicate that HpETEs can interact with Met_93_. The side-chain of Met_157_ is buried and a lack of interaction between this residue and L-OOHs is therefore expected. However, Met_49_ is relatively exposed in the crystal structure (Figure 2A) and was observed to interact with solvent in the simulations described above. It was therefore unclear why the interaction of HpETEs with apoD do not generate Met_49_SO. Given the location of N-glycan structures at Asn_45_ and Asn_78_ [25], we hypothesized that the N-glycan moiety at Asn_45_ might shield Met_49_ and prevent L-OOH mediated Met oxidation. We therefore ran an extended MD simulation of glycosylated apoD. The N-glycan structures present at Asn_45_ and Asn_78_ have been previously characterized and found to be mostly represented by trisialo triantennary and fucosylated disialo biantennary oligosaccharides, respectively [25], as graphically represented in Figure 7A. The conformations of the N-glycan chains are represented in Figure 7B. Clearly, access to Met_49_ is partially blocked by the trisialo triantennary N-glycan at Asn_45_ whereas neither of the N-glycans obstruct access to the apoD ligand binding pocket (Figure 7B). This provides a plausible explanation for the previously reported lack of reactivity between the apoD Met_49_ side chain and various HpETEs [15].

### CD analysis indicates MetSO formation does not induce major structural changes

**Figure 8:**
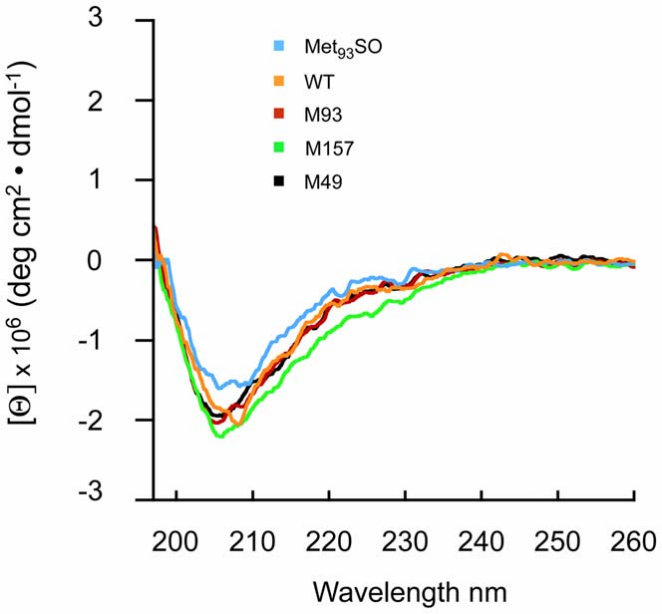
CD spectra of recombinant apoD. Far-UV CD spectra of apoD and apoD-Met_93_SO and the Met to Ala mutants indicated. Spectra were obtained in PBS, pH 7.4 at 22°C.

The above simulations give a plausible explanation for the selective oxidation of Met_93_ by L-OOHs. Based on this information and on our previous observations that the formation of Met_93_SO promotes apoD self-association and aggregation [15], we went on to investigate the possible structural changes that may be induced by apoD Met_93_SO formation. The CD spectrum of recombinant wild type apoD and all three Met to Ala mutants shows a negative minima in ellipticity at 208 nm (Figure 8) and overall is in very close agreement with previous studies [26]. Furthermore, the CD spectrum of HpETE-treated apoD (previously shown to convert apoD Met_93_ to Met_93_SO, [15]) was very similar to both the wild type apoD and the Met to Ala mutants (Figure 8). This indicates that neither mutation of apoD Met residues to Ala nor conversion of Met_93_ to Met_93_SO induce major changes to apoD secondary structure and, in particular, that the β-barrel structure remains in tact.

### MD simulaltion of apoD and apoD-Met_93_SO shows increased mobility in response to Met_93_ oxidation

**Figure 9:**
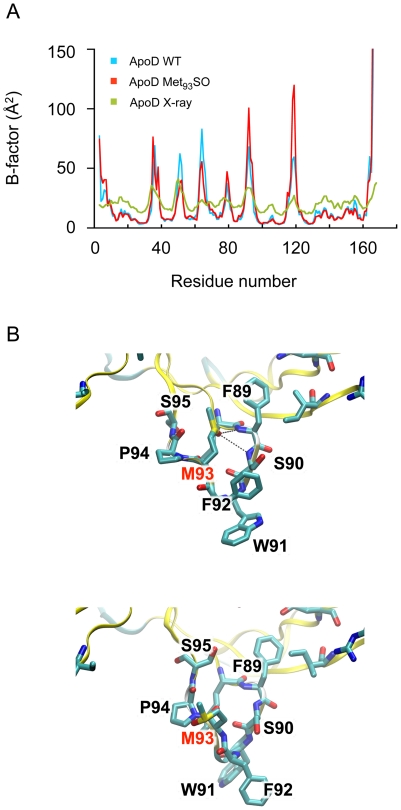
MD simulation of apoD and apoD-Met_93_SO. (*A*) B-factors calculated from the MD simulations of apoD, apoD-Met_93_SO and crystallographic B-factors are shown. (*B*) Snapshots from the apoD-Met_93_SO simulation. Initially the sulfoxide oxygen of Met_93_SO remains in the initially modelled conformation, similar to Met93 in the ligand-free crystal structure. Simulation of apoD-Met_93_SO suggested that the sulfoxide oxygen of Met_93_SO could accept hydrogen bonds from the amide-nitrogen groups of Phe_89_ and Ser_90_. (upper structure). After 7 ns, the side-chain flips out of the pocket and is surrounded by water molecules. A representative snapshot of the populations of side-chain conformations is shown (lower structure).

Because no major changes in apoD secondary structure were detected by CD spectroscopy, we next used MD simulations to probe for structural changes that may be induced by apoD Met_93_SO formation. ApoD and apoD-Met_93_SO were simulated for 50 ns. The proteins were stable over that timeframe. In both simulations the RMSD of Cα atoms initially increased and stabilized at around 2–2.5 Å (data not shown). Calculated B-factors from the simulations matched the pattern of B-factors in the X-ray structure (PDB: 2HZQ) (Figure 9A). The B-factors of the loop containing Met_93_SO were higher in the simulation of the oxidized protein, indicating increased flexibility. In the apoD simulation, Met_93_ remained associated with the side-chains of Phe_89_ and Phe_92_ via hydrophobic interactions. In the apoD-Met_93_SO simulation, the side-chain displayed more flexibility. Initially, the side-chain sulfoxide oxygen atom accepted hydrogen bonds from the main-chain amino groups of Phe_89_ and Ser_90_. After 7 ns, the Met_93_SO side-chain flipped out of the pocket and transiently associated with the side-chains of Phe_89_ and Phe_92_ in a similar fashion to native Met_93_. For the last 30 ns of the simulation, the side-chain of the oxidized residue was observed to be surrounded by water molecules and not directly interacting with other residues. A representative snapshot of this flipped-out conformation is shown in Figure 9B. The increase in the mobility of the Met_93_SO side chain that we have identified increases the thermal motion of the loop in which it is incorporated and this is predicted to destabilize apoD structure.

## Discussion

The results of the simulation of apoD with progesterone give us confidence that the CHARMM force-field is sufficiently accurate to investigate the interactions of this protein with representative ligands. The simulations of apoD with arachidonate indicate that this ligand has considerable flexibility. This is consistent with the structural plasticity of the fatty acid and the broad, open nature of the apoD binding site, which is ∼15 Å deep and 10 Å×15 Å wide at the entrance. It is noteworthy that the low-energy conformations of arachidonate used for the modeling of L-OOHs is similar to the mode of binding proposed by Eichinger and co-workers [1]. Although the simulations of apoD with arachidonate totaled 800 ns, it may be argued that this timeframe is insufficient for the system to converge on a global minimum, and that the optimum binding pose found in nature was not sampled in our simulations. However, even if such a state exists, fatty acids would be expected to explore a large number of conformations (such as those presented here) prior to converging on a hypothetical low-energy state. Consistent with our experimental results [15], our modeling indicate that the oxygenated fatty acid products of 5-, 12- and 15-lipoxygenase bind at the entrance of the ligand binding pocket of apoD in configurations that would permit direct interaction between the lipid hydroperoxide moiety and the crucial Met_93_ side chain. This interaction occurs as these highly flexible compounds can slide within the binding-site with many conformations of similar energy available.

Our data suggest that the solvent-exposed Met_93_, located in the hydrophobic region at the opening of the apoD ligand-binding pocket may also interact with lipid membrane surfaces from which phospholipid-bound fatty acid hydroperoxides protrude due to the addition of the polar peroxide group [27]. The side-chain of Met_157_ is buried and therefore not expected to be solvent exposed to lipid hydroperoxides. However, Met_49_ is solvent exposed and therefore has potential to interact with L-OOHs. The simulation of apoD with physiologically relevant glycosylation suggests that the ∼5 kDa N-linked oligosaccharide at Asn_45_ could have a shielding effect on Met_49_. From our simulation data, this oligosaccharide is also expected to sterically preclude apoD from approaching a membrane in an orientation favouring interaction with Met_49_. Glycosylation may therefore be one of the mechanisms by which aberrant oxidation of apoD Met_49_ is prevented.

It is noteworthy that apoD Met_93_ is highly conserved in mammals (Figure 1) and we speculate that this may afford an evolutionary advantage in terms of providing a lipid antioxidant function in the brain and possibly a protective function for the apoD ligand-binding pocket itself. Relevant to this latter point, the high propensity for redox-active Met residues to be located in close proximity to protein ligand binding sites/functional domains has previously been suggested to confer localized “guardian” antioxidant protection [28].

The mechanism underlying the increase in apoD aggregation that is associated with Met-SO formation [15] is not entirely clear. Previous research indicates that even though the introduction of an oxygen atom in the Met side chain would be expected to decrease the hydrophobicity of the protein (consistent with the decrease in reversed phase HPLC RT we have reported previously [15]), this modification can induce structural changes in the protein that increase the exposure of hydrophobic residues [29], [30]. This prediction is consistent with our simulation of apoD-Met_93_SO that suggested the sulfoxide oxygen could initially accept hydrogen bonds from the amide-nitrogen groups of Phe_89_ and Ser_90_ and then flip out of the entrance to the binding pocket to transiently associate with the side-chains of Phe_89_ and Phe_92_ and then adopt a conformation surrounded by water molecules without interacting with other residues. Based on previous studies [31], this may promote local unfolding of the protein structure and increase the propensity for apoD to self-associate and aggregate.

Previous studies have reported that the destabilization of protein structure as a consequence of MetSO formation can have profound functional consequences. The oxidation of p53 Met_340_ residue located within the hydrophobic core of the p53 tetramer significantly reduces p53 stability and is thought to represent one inactivation mechanism for p53 transcriptional function [32]. Interestingly, MD simulations assessing the oxidation of Met_213_ within Helix-3 of the cellular prion protein (PrP^C^) indicated a destabilization of the PrP^C^-like fold that favors more flexible states that are prone to conformation transition into the pathogenic infectious prion (PrP^Sc^) form [33]. Furthermore, mutations of the PrP^C^ that are associated with familial prion disorders increase the solvent exposure of Met_213_ and thus increase the probability for MetSO formation [34]. More directly related to the current study, previous research has shown that plasma apolipoprotein A-I (apoA-I) also has the capacity to promote the conversion of L-OOHs to L-OHs via a 2-electron redox reaction that concomitantly converts apoA-I Met residues to their respective MetSO moieties [35], [36]. Recent studies have shown that oxidation of apoA-I Met residues caused a partial unfolding of the protein, decreased its thermal stability and promoted its fibrillization [37]. It therefore appears that protein destabilization induced by selective Met oxidation may be common to several pathogenic processes.

In summary, our MD simulations provide a plausible mechanism that helps to explain how different forms of HpETEs can selectively interact with apoD Met_93_ and also reveal a potential basis for the destabilization of and self-association of apoD structure that results from Met_93_SO formation.
